# Ginseng Berry Juice (GBJ) Regulates the Inflammation in Acute Ulcerative Mouse Models and the Major Bioactive Substances Are Ginsenosides Rb3, Rc, Rd, and Re

**DOI:** 10.3390/nu16071031

**Published:** 2024-04-01

**Authors:** Soon-Young Lee, Seung-Yub Song, Sung-Ho Lee, Gye-Yeop Kim, Jin-Woo Park, Chun-Sik Bae, Dae-Hun Park, Seung-Sik Cho

**Affiliations:** 1 College of Oriental Medicine, Dongshin University, Naju-si 58245, Republic of Korea; 2 Department of Pharmacy, College of Pharmacy, Mokpo National University, Muan-gun 58554, Republic of Koreajwpark@mnu.ac.kr (J.-W.P.); 3 Biomedicine, Health and Life Convergence Sciences, BK21 Four, College of Pharmacy, Mokpo National University, Muan-gun 58554, Republic of Korea; 4 Department of Physical Therapy, Dongshin University, Naju-si 58245, Republic of Korea; 5 College of Veterinary Medicine, Chonnam National University, 77 Yongbong-Ro, Buk-Gu, Gwangju 61186, Republic of Korea; csbae210@chonnam.ac.kr

**Keywords:** ginseng berry juice, acute inflammation, ginsenosides Rb3, Rc, Rd, and Re

## Abstract

*Panax ginseng* fruit is known to have various biological effects owing to its large amount of saponins such as ginsenosides. In the present study, ginseng berry juice was confirmed to be effective against acute inflammation. Ginseng berry juice was used for analysis of active constituents, antioxidant efficacy, and *in vivo* inflammation. A high-performance liquid chromatography method was used for analysis of ginsenosides. In an HCl/ethanol-induced acute gastric injury model, microscopic, immunofluorescent, and immunohistochemical techniques were used for analysis of inhibition of gastric injury and mechanism study. In a mouse model of acute gastritis induced with HCl/ethanol, ginseng berry juice (GBJ, 250 mg/kg) showed similar gastric injury inhibitory effects as cabbage water extract (CB, 500 mg/kg, P.O). GBJ dose-dependently modulated the pro-inflammatory cytokines such as Tumor Necrosis Factor-α (TNF-α), Interleukin-6 (IL-6), and Interleukin-13 (IL-13). GBJ inhibited the activation of Nuclear Factor kappa bB (NF-κB) and suppressed the expressions of cyclooxigenase-2 (COX-2) and prostaglandin 2 (PGE_2_). The anti-inflammatory effect of GBJ is attributed to ginsenosides which have anti-inflammatory effects. Productivity as an effective food source for acute gastritis was analyzed and showed that GBJ was superior to CB. In addition, as a functional food for suppressing acute ulcerative symptoms, it was thought that the efficacy of gastric protection products would be higher if GBJ were produced in the form of juice rather than through various extraction methods.

## 1. Introduction

Ginseng berry (GB) is a *Panax ginseng* (PG) fruit. Generally, the root of PG is used for therapeutic purposes. However, GB fruit also contains various pharmacological components such as ginsenoside, and because the method for obtaining the fruit extract is simple, many industrial studies on GB fruit extract are being conducted. Representative biological effects of GB include anti-obesity [1,2], anti-inflammatory [3,4], neuroprotective [5], hepato-protective [6], and skin whitening [7].

GB, except for the seeds, is edible and can be developed as a therapeutic or preventive agent for metabolic diseases [1,2,3,4,5,6]. In addition, its extract is easy to separate and dry. In particular, when developing as a functional food, it is more advantageous than the PG roots or stems, because of its low unit cost and easy processing. Although research based on the function of GB has been continuously conducted, information on the optimal extraction process, index selection, and production standardization is lacking.

Xi et al. confirmed that oral administration of 0.6 mL/kg of American GB juice daily to ob/ob mice for 10 days lowered fasting blood glucose. Xi et al. prepared 200 mL of juice with 150 g of GB (750 mg/mL). It has been reported that intake of GB as fruit can help control blood glucose [8]. Xi et al. also analyzed the ginsenoside content of GB extract and reported that the total ginsenoside content was 5.46 mg/mL. The specific amounts were: ginsenosides Rg1 (0.04 mg/mL), Re (0.88 mg/mL), Rh1 (0.003 mg/mL), Rg2 (0.06 mg/mL), 20R-Rg2 (0.02 mg/mL), Rb1 (0.05 mg/mL), Rb2 (0.77 mg/mL), Rb2 (2.9 mg/mL), Rd (0.39 mg/mL), Rg3 (0.03 mg/mL), Rh2 (0.02 mg/mL), and the major biomarker was Rb2.

In a study on memory improvement, HU et al. reported memory improvement effects of GB extracts and tacrine. HU et al. reported that the GB extract was superior to tacrine (10 mg/kg) in the regulation of GSH levels, SOD activity, ChAT, PI-3K, Akt, and ERK2 mRNA expression when the GB extract was administered at 400 mg/kg, and its anti-amnesic efficacy was also superior to those of tacrine. However, the anti-amnesic effect of GBE (200–400 mg/kg) was similar to that of tacrine (10 mg/kg). HU et al. demonstrated that GB extract was effective in preventing various neurodegenerative diseases accompanied by memory impairment, and the human dose for prophylaxis is estimated to be at least 975 mg/60 kg human [9]. According to Kim et al., GB extract was prepared in powder form by extracting GB at 80 °C for 10 h; the powder form was preferred because it could be easily prepared as a hot water extract. Although the extraction yield is not described in their report, it is thought that it can be developed as a competitive memory-improving functional material through optimization of hot water extraction, standardization of analysis, and process improvement. In particular, the human dose is expected to be 1–2 g, thus development as a supplementary material is likely to be advantageous [10].

Nam et al. reported the hepato-protective effect of GB extract (GBE) in a rat mild bile duct ligation model. A GB extract was prepared by ultrasonication, and silymarin (150 mg/kg) was used as a positive control. After performing mild bile duct ligation in rats, they orally administered ultrasonicated GBE (100 mg/kg, 250 mg/kg, 500 mg/kg), GBE (250 mg/kg), and silymarin (150 mg/kg) for six weeks. Nam et al. reported that ultrasonicated GBE inhibited hepatic fibrosis by inhibiting the TLR4 signaling pathway in the liver in a dose-dependent manner. Ultrasonicated GBE was made by treating 4-year-old dried ginseng fruit with ethanol to make an extract (GBE), followed by ultrasonication for 10 h. The GBE contained ultrasonication-degraded ginsenoside Re, Rb2, and Rf, and Rg3, Rh4, Rk1, and Rk3 were also discovered [6]. In particular, ginsenoside Re, the main substance of GBE, is almost completely removed. As a result, there is no main biomarker in ultrasonicated GBE, which is a limitation in that there is no key compound that can prove the mechanism of pharmacological effect. To develop ultrasonicated BGE as a pharmaceutical material in the future, further research is needed, such as studies on optimal manufacturing processes and selection of biomarkers.

Shin et al. reported that GB extract is effective against sarcopenic obesity induced by a high-fat diet and that it reduces food efficiency ratio, serum lipid and insulin levels, adipose tissue weights and adipocyte size, grip strength, and muscle masses, and increases the myofiber cross-sectional area. The optimal dose of GB extract was 100–200 mg/kg, and the human dose was calculated as 487–975 mg/60 kg/day.

Shin et al. made a hot water extract powder from a 4-year-old GB by a standardized method. The yield was 2.5% and standardized to contain 5% of ginsenoside Re as the major component, and six ginsenosides, which accounted for 15.19% of the total ginsenosides (0.45%, 0.90%, 1.11%, 0.75%, 6.06%, 1.16%, and 0.53% for Rb1, Rb2, Rc, Rd, Re, Rg1, and Rg2, respectively) [11]. Thus, Shin et al. presented standardized manufacturing methods, biomarkers, and standard test methods appropriately, and the optimal dose of GB extract for preventing sarcopenic obesity was proposed.

The GB-related studies reported so far have clearly highlighted pharmacological activity, but studies for the application of preventive sources and functional food sources are poor. In a previous study, we reported the results of changes in physiological effects and biomarkers according to the optimal harvest time point of GB [12]. In this study, we removed the seeds of GB and prepared it as juice (GBJ). We confirmed the effect of oral administration of GB juice (GBJ) on acute gastric ulcers, presented the efficacy mechanism and optimal administration dose, and attempted to derive the optimal dose that can be administered once to humans. In addition, we sought to determine whether consuming GB as juice is scientifically preferable for acute gastric ulcers through the inhibition of gastric damage, mechanism of action, and relationship with biomarkers.

## 2. Materials and Methods

### 2.1. Plant Materials and Analytic Conditions

Ginseng berry from 4-year-old ginseng (1 kg) was harvested from a local farm (Healthy Sam-Farm, Iksan, Republic of Korea). GB seeds were removed and juiced (GBJ). The juice was freeze-dried and the yield was 59%. GBJ was stored at −70 °C before the experiment. Biomarkers in GBJ were performed according to the previously reported method [12]. Cabbage (*Brassica oleracea* var. capitata L.) hot water extract (CB) was used as a control. CB was supplied by Green Food (Jeonnam, Republic of Korea). Cabbage (1 kg) was extracted with hot water for four hours and the aqueous phase was freeze-dried, and the yield was 12%. Analytical standards (Sigma–Aldrich Co., St. Louis, MO, USA) were used for HPLC analysis of biomarker concentrations in GB.

### 2.2. Analysis of Biomarkers

All analyses were performed on a Waters Alliance 2695 HPLC (Waters Co., Milford, MA, USA) system with a photodiode array detector. The analytical column used was an Agilent Zorbax extended C18 (5 μm, 150 mm × 5 mm, Agilent Tech, Santa Clara, CA, USA), with the mobile phase consisting of a mixture of acetonitrile (A) and 0.2% phosphoric acid (B) and employing a gradient elution (from 10/90 to 100/0, *v*/*v*) at a flow rate of 0.8 mL/min. The column temperature was maintained at 25 °C, and the detection wavelength was set at 210 nm. Injection volume was 20 μL (Table 1).

### 2.3. HCl/Ethanol-Induced Acute Gastric Injury Model

Male 7-week ICR mice (n = 6 each group) were purchased from Samtako Korea (Osan, Republic of Korea). Before the study, all the animals were acclimated for seven days, and were fasted for 12 h before agent administration, including tap water for the control group. Animal groups consisted of the control group (tap water treatment), positive control group (500 mg/kg CB administration), and 3-GBJ-dose administration groups (50 mg/kg, 250 mg/kg, or 500 mg/kg), and all agents including tap water were orally administered. At 1 h after all the agents’ administration, the groups were treated with 500 μL 150 mM HCl and 60% ethanol, excluding the control group, for one hour.

### 2.4. Macroscopic and Microscopic Analysis

To evaluate the anti-gastric ulcerative effect of GBJ on gastric injury, after all agents’ administration, animals were euthanized using 50 mg/kg Zoletin (Virbac, Fort Worth, TX, USA). For macroscopic measurement, the stomachs were excised and photographed on the damaged area, and these areas were measured using the ImageJ program. The gastric damage inhibition ratio was calculated by the following equation.

Gastric damage inhibition rate (%) = (Area (control) − Area (sample))/Area (control) × 100 (Area: mm^2^)

For microscopic evaluation, the stomachs were fixed in 10% formaldehyde solution (*v*/*v*) at room temperature for 1 month, dehydrated in ethanol (99.9, 90, 80, and 70%), and embedded in paraffin. The paraffin-embedded tissues were sectioned (5 µm) and stained with hematoxylin and eosin (H&E). The results were obtained with an Axioscope A1 microscope (Carl Zeiss AG, Jena, Germany) [13]. After H&E staining, the level of tissue’s change was classified based on the criteria of the histopathological score. Score 0 means no inflammation in the mucous membrane or submucosa, and as the score increases to 5, neutrophil and macrophage infiltration into the mucosa, submucosa, and muscle layer, and extensive necrosis, edema, erosion, and ulceration progressed in all parts of the gastric epithelium.

### 2.5. Immunofluorescent and Immunohistochemical Analyses

After deparaffinizing the tissues, 3% hydrogen peroxide in methanol was added and the tissues were left for 10 min to remove the endogenous peroxidase, and the antigen retrieval step was done in 0.1 M sodium citrate buffer on a hot plate. After preventing the nonspecific binding normal serum step, the primary antibody binding step was done for 1 h at 4 °C such as TNF-α (MBS175453, MY BioSource, San Diego, CA, USA), IL-6 (sc-7920, Santa Cruz, TX, USA), IL-13 (sc-1776, Santa Cruz), IL-10 (sc-73309, Santa Cruz), NF-κB (51-0500, Invitrogen, Carlsbad, CA, USA), COX-2 (ab15191, Abcam, Boston, MA, USA), and PGE_2_ (bs-2639R, Bioss, Woburn, MA, USA). To detect NF-κB protein or the nucleus, immunofluorescent analysis was done with Alexa Fluor 488-conjugated secondary antibody (A3273, Invitrogen) and DAPI (62249, ThermoFisher Scientific, Waltham, MA, USA), respectively, and the results were acquired using a K1-Fluo confocal microscope (Nanoscope System, Daejeon, Republic of Korea). However, for measuring COX-2 protein or PGE_2_, one immunohistochemical analysis was conducted after binding pan-specific secondary antibody, and all sectioned tissues were treated with streptavidin peroxidase (Vector Laboratories Universal Quick Kit, Burlingame, CA, USA).

### 2.6. Ethics Statement

Animal study was conducted according to guidelines of the Institutional Animal Care and Use Committee at the Chonnam National University (Approval No. CNU IACUC-YB-R-2019-49).

### 2.7. Statistical Analysis

Results were expressed as mean ± standard deviation (SD). Group differences were evaluated by one-way analysis of variance and followed by Dunnett’s multiple comparison test. Significance was considered at *p* < 0.05.

## 3. Results

### 3.1. GBJ Prevented the HCl/Ethanol-Induced Morphological Changes

**Figure 1:**
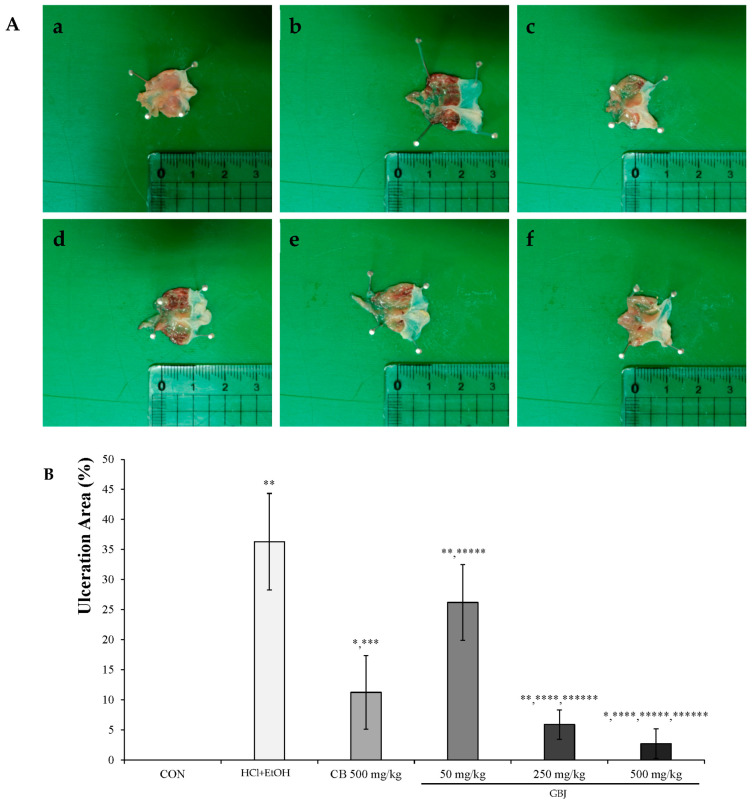
Anti-gastritis effect of GBJ in HCl-ethanol murine model. (**A**) GBJ dose-dependently HCl-ethanol-induced gastritis. HCl-ethanol treatment induced chemical burning but GBJ prevented that in a dose-dependent manner. (**B**) GBJ dose-dependently suppressed the HCl-ethanol-induced ulcerative area. a, control; b, HCl-ethanol-induced gastritis group; c, 500 mg/kg CB administration group; d, 50 mg/kg GBJ administration group; e, 250 mg/kg GBJ administration group; f, 500 mg/kg GBJ administration group. The unit-of-scale bar is in centimeters. The results were expressed as mean and standard deviation. * *p* < 0.05 vs. CON; ** *p* < 0.001 vs. CON; *** *p* < 0.05 vs. HCl-EtOH-induced gastritis group; **** *p* < 0.001 vs. HCl-EtOH-induced gastritis group; ***** *p* < 0.05 vs. 500 mg/kg CB administration group; ****** *p* < 0.001 vs. 50 mg/kg GBJ administration group.

GBJ dose-dependently prevented the HCl/ethanol-induced gastric injury, and in the 250 mg/kg GBJ administration group, the inhibition rate against gastric damage was about 80% compared to 70% preventing effect in the 500 mg/kg CB administration group, which was a positive control group. Notably, the rate in the 500 mg/kg GB administration group reached 90%. GBJ was thought to not only have a dose-dependent preventing effect against acute chemical-induced gastric damage but also to be more effective than the positive control group (Figure 1A). The gastric ulcer area was 7 ± 2.45 mm^2^ in the 250 mg/kg GBJ administration group, which was 50% compared to the 500 mg/kg CB treatment group (10 ± 6.13 mm^2^), which is a positive control group. In the 500 mg/kg GBJ treatment group, the gastric ulcer area was 2.5 ± 2.47 mm^2^. Taken together, GB was thought to effectively suppress the occurrence of gastric ulcer (Figure 1B).

### 3.2. BGJ Prevented the HCl/Ethanol-Induced Histopathological Changes

**Figure 2:**
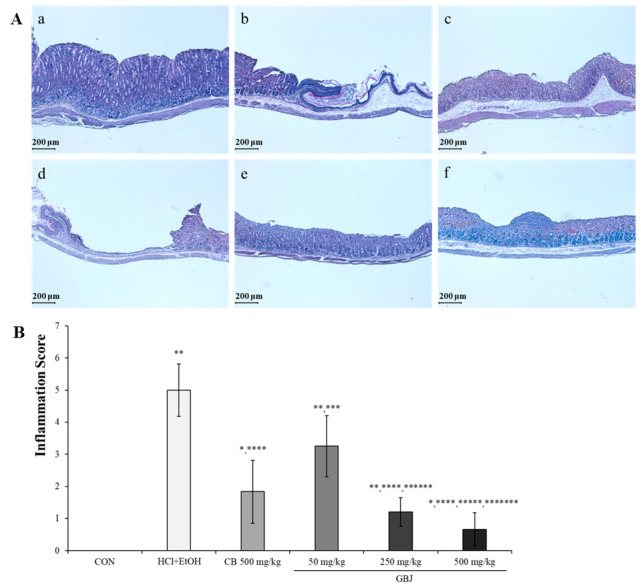
Anti-ulcerative effect of GBJ in histopathological analysis. (**A**) GBJ prevented HCl-ethanol-induced stomach ulcer in a dose-dependent manner. a, control; b, HCl-ethanol-induced gastritis group; c, 500 mg/kg CB administration group; d, 50 mg/kg GBJ administration group; e, 250 mg/kg GBJ administration group; f, 500 mg/kg GBJ administration group. Scale bar, 200 μm. Magnification, ×200. (**B**) GBJ dose-dependently suppressed HCl-ethanol-induced inflammation. The results were expressed as mean and standard deviation. * *p* < 0.05 vs. CON; ** *p* < 0.001 vs. CON; *** *p* < 0.05 vs. HCl-EtOH-induced gastritis group; **** *p* < 0.001 vs. HCl-EtOH-induced gastritis group; ***** *p* < 0.05 vs. 500 mg/kg CB administration group; ****** *p* < 0.05 vs. 50 mg/kg GBJ administration group; ******* *p* < 0.001 vs. 50 mg/kg GBJ administration group.

In order to evaluate the level of HCl-ethanol-induced gastric ulcer, H&E stain was performed (Figure 2A). HCl-ethanol administration induced the damage to the mucous layer of the stomach (Figure 2(Ab)), but 500 mg/kg CB administration prevented the gastric ulcer (Figure 2(Ac)). Although in the 50 mg/kg GBJ treatment group HCl-ethanol-induced gastric damage could not be prevented, (Figure 2(Ad)) in both the 250 mg/kg GBJ treatment group and the 500 mg/kg GBJ treatment group, the gastric ulcer was completely prevented (Figure 2(Ae,f)). The H&E-stained tissue was visually assigned an inflammation score to evaluate the severity of tissue damage between each group (Figure 2B). Inflammation inhibition by GBJ was approximately 1.5 in the 250 mg/kg administration group compared to the 500 mg/kg CB administration group (2 ± 0.98), which is the positive control group. In the 500 mg/kg GBJ administration group, it was 1, which is 50% dosage of the positive control group (Figure 2B). Therefore, it is thought that GBJ was more effective in inhibiting gastric inflammation than CB as a positive control drug.

### 3.3. GBJ Suppressed the Expression of Pro-Inflammatory Cytokines Such as TNF-α, IL-6, and IL-13 but Increased the Expression of Anti-Inflammatory Cytokines Such as IL-10

**Figure 3:**
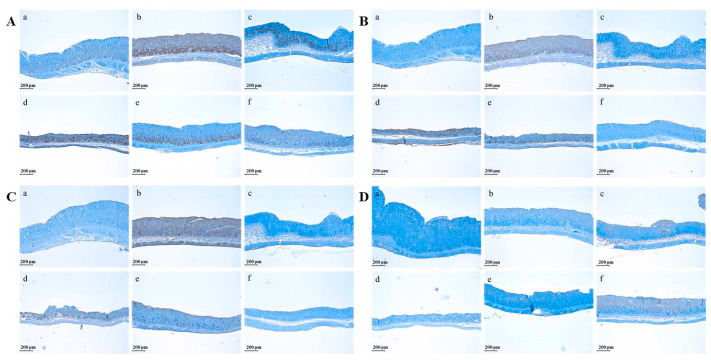
Anti-inflammatory effect of GBJ. GBJ administration dose-dependently inhibited the pro-inflammatory cytokine such as (**A**) TNF-α, (**B**) IL-6, and (**C**) IL-13 but stimulated the expression of anti-inflammatory cytokine such as (**D**) IL-10 in a dose-dependent manner. a, control; b, HCl-ethanol-induced gastritis group; c, 500 mg/kg CB administration group; d, 50 mg/kg GBJ administration group; e, 250 mg/kg GBJ administration group; f, 500 mg/kg GBJ administration group. Scale bar, 200 μm. Magnification, ×200.

GBJ controlled the expression of pro-inflammatory cytokines such as Tumor Necrosis Factor-α (TNF-α), Interleukin-6 (IL-6), and Interleukin-13 (IL-13) and promoted the production of Interleukin-10 (IL-10) (Figure 3). HCl/ethanol treatment stimulated the expressions of TNF-α, IL-6, and IL-13 in the gastric mucosa (Figure 3(Ab,Bb,Cb)), while CB inhibited the expression levels of IL-6 and IL-13 (Figure 3(Bc,Cc)). However, the suppression level of TNF-α expression by CB was somewhat weak compared to two cytokines such as IL-6 and IL-13. GBJ dose-dependently suppressed the expression of TNF-α, IL-6, and IL-13 (Figure 3(Ad,e,Bd,e,Cd,e)). In addition, in the 500 mg/kg GBJ-treated group, the expression levels of all pro-inflammatory cytokines such as TNF-α, IL-6, and IL-13 were down-regulated compared to the CB-treated group (Figure 3(Ae,Be,Ce)). However, in the case of anti-inflammatory cytokine IL-10, GBJ treatment increased its expression in a dose-dependent manner (Figure 3D).

### 3.4. GBJ Controls the Translocation of NF-κBp65 from the Cytoplasm to the Nucleus to Prevent Inflammation and Then Suppresses the Production of Cyclooxygenase 2 (COX-2) and Prostaglandin E_2_

**Figure 4:**
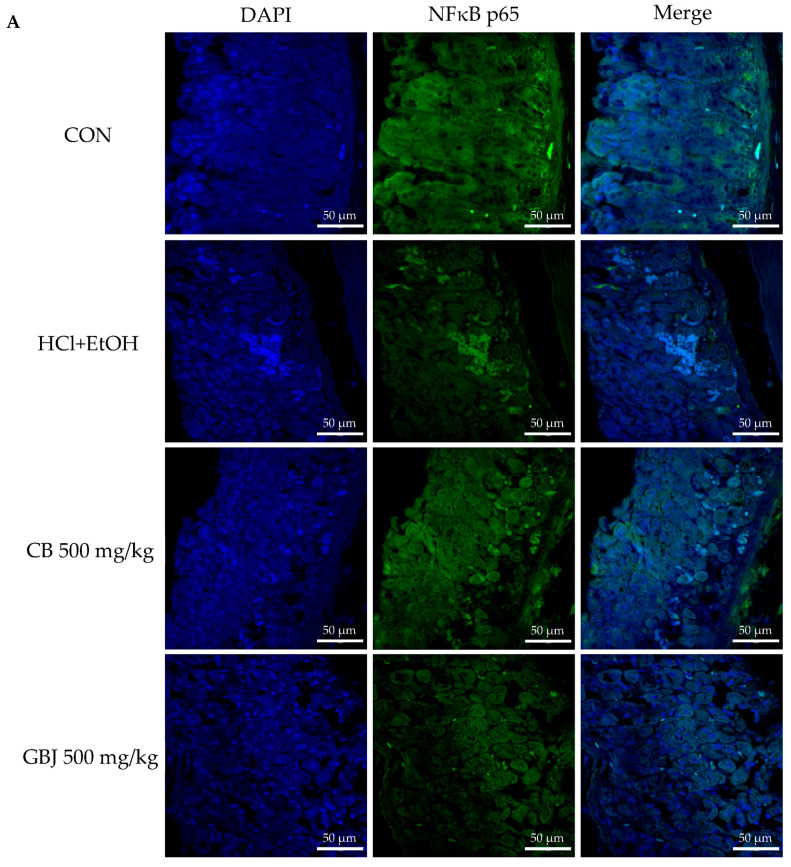

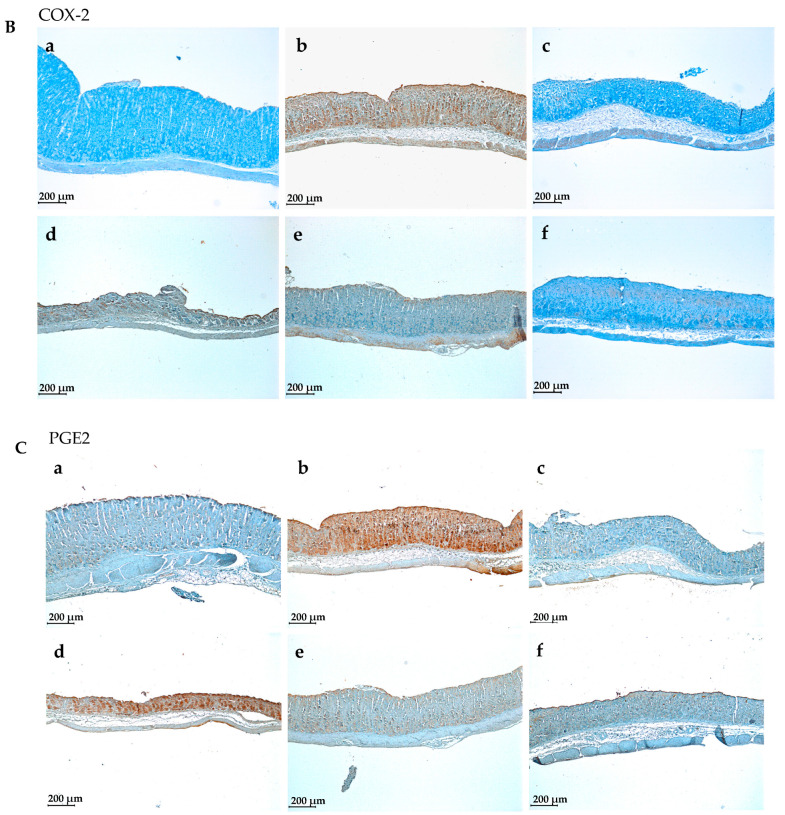
GBJ suppressed the inflammation occurrence through NF-κB/COX-2/PGE_2_ pathway. (**A**) GBJ administration inhibited HCl-ethanol-induced NF-κB translocation from the cytoplasm to the nucleus and then NF-κB was inactivated. Scale bar, 50 μm. Magnification, ×400. GBJ dose-dependently inhibited not only (**B**) the COX-2 expression but also (**C**) PGE_2_ expression. a, control; b, HCl-ethanol-induced gastritis group; c, 500 mg/kg CB administration group; d, 50 mg/kg GBJ administration group; e, 250 mg/kg GBJ administration group; f, 500 mg/kg GBJ administration group. Scale bar, 200 μm. Magnification, ×200.

HCl/ethanol treatment-induced inflammation occurred through the activation of tumor necrosis factor kappa B (NF-κB) and the increment of expressions of cyclooxygenase 2 (COX-2) and prostaglandin E_2_ (PGE_2_) (Figure 4). Compared to the results of CB treatment on the NF-κB activation (translocation from the cytoplasm to the nucleus), 500 mg/kg GBJ treatment significantly controlled the activation of NF-κB (Figure 4A). Similar to the results of NF-κB expression, GBJ treatment suppressed the expressions of COX-2 and PGE_2_ and dose-dependently inhibited the levels of COX-2 protein and PGE_2_. (Figure 4Bd–f,Cd–f). In a previous study, the ginsenoside Re also blocked the expression of COX-2, PGE2, and the activation of NF-κB, indicating that ginsenoside Re could be one of the major anti-inflammatory saponins present in GBJ.

### 3.5. Preparation and Quantitative Analysis of Anti-Ulcerative Biomarkers in GBJ

In the present study, it was confirmed *in vivo* that GBJ was independently effective in acute gastritis. In this study, for GBJ, the seeds of GB were removed, the juice was filtered and then freeze-dried, and the yield was 59.01%. Since cabbage, a control, is commercially sold as a hot water extract, we used the freeze-dried hot water extract and the yield was 12%. In the HCl/ethanol-induced inflammation mouse model, GBJ showed anti-inflammatory efficacy at the same or a lower dose (250, 500 mg/kg) than CB (500 mg/kg); thus, GBJ is considered to be a beneficial source in terms of biological effects and productivity.

**Figure 5:**
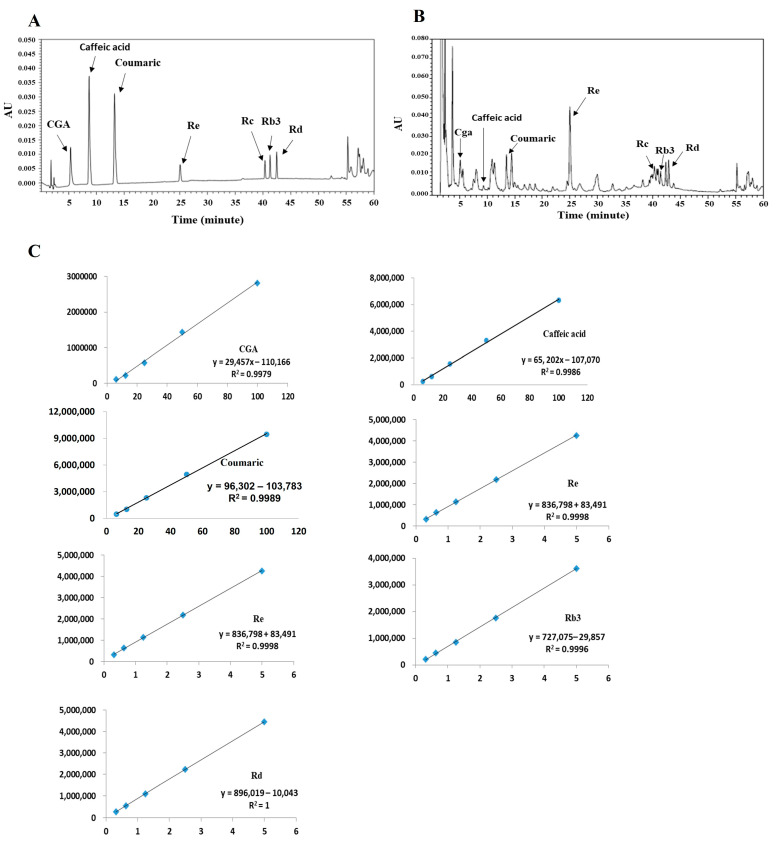
HPLC profiles of GBJ. (**A**) Standards, (**B**) GBJ, (**C**) Calibration curves. All standards were used for analysis through a standard curve.

Furthermore, we confirmed that GBJ contains several biomarkers (Figure 5). The contents of ginsenosides Rb3, Rc, Rd, and Re in GBJ were 2.55 ± 0.04%, 2.12 ± 0.16%, 2.73 ± 0.02%, and 5.21 ± 0.21% (*w*/*w*), respectively. Ginsenoside Re accounted for the largest amount, and the contents of ginsenosides Rb3, Rc, and Rd were 2–3%. The total content of analyzed ginsenoside was about 12.5% and is considered to be the main biomarker of GBJ. A trace amount of biologically active substances such as chlorogenic acid, caffeic acid, and coumaric acid were also identified in GBJ, and were contained within 0.25% (Table 2).

## 4. Discussion

*Panax ginseng* has been one of the most popular food sources for health care and disease treatment in Asia, including Korea and China, and is currently distributed in 35 countries around the world. *P. ginseng* can be used for medicines, functional food, and agricultural products. In general, South Korea, China, Canada, and the US are the biggest producers and their total production of fresh ginseng is approximately 79,769 tons, which is more than 99% of the 80,080-ton total ginseng production around the world [14]. *Panax ginseng* berry is a by-product of ginseng. As ginseng production increases, the amount of by-product increases, and consequently, there are ongoing research efforts to find effective ways to utilize the berry as well.

Several biological effects of *P. ginseng* berry and its active compounds have been reported. Kim et al. reported that acidic-polysaccharide-linked glycopeptide derived from *P. ginseng* berry exerted an anti-immunosenescent effect by suppressing thymic involution and modulating several types of immune cells. The anti-immunosenescent effect [15] was demonstrated by oral administration to mice at a dose of 30 mg/kg, which is equivalent to 146 mg/60 kg administered to humans. However, Kim et al. did not describe the extraction yield of polysaccharides. Thus, the effective daily dose of ginseng berry could not be calculated.

Nam et al. reported that ultrasonicated ginseng berry extract showed a hepatoprotective effect in the rat liver injury model and mild bile duct ligation model. They reported that the hepatoprotective effect in rats was observed from 100 mg/kg [6,16]. The hepatoprotective effect was shown by oral administration to rats at a dose of 100 mg/kg, which is equivalent to 972 mg/60 kg administered to humans. However, Nam et al. did not describe the extraction yield of the extract and could not calculate the effective daily dose of ginseng berry. They suggested that ginsenosides Rg2, Rk1, Rg3, and Rh1 were effective markers of their source because these compounds were shown to have liver antioxidant [17], anti-inflammatory [18], and anti-carcinogenic effects [19].

The mechanism of action of ginsenoside has been described in vitro (cell level), but its efficacy in animals and the human body has not been determined. If the optimal dose was determined by animal experiments in the previous reports, the appropriate dose in humans could be predicted.

Therefore, based on the report of Nam et al., we could deduce the human dose of ginseng berry extract, but could not conclude that the four ginsenosides are key compounds for hepatoprotective effect. In the previous report, Nam et al. reported that the contents of ginsenosides Rg2, Rg3, Rh1, and Rk1 in ultrasonicated extract were 2.28%, 0.83%, 1.35%, and 0.2%, respectively. They reported that the content of Re in the general extract was 11.17% and the content of Re in the ultrasonicated extract decreased to 0.28%. Therefore, it was thought that Re was not a liver protection indicator. As the content of ginsenosides Rg2, Rg3, Rh1, and Rk1 increased, they were predicted to induce liver injury in the bile duct ligation rat model. Therefore, future studies should evaluate the efficacy of the four active compounds (Rg2, Rg3, Rh1, and Rk1), and characterize and standardize the extraction process for the development of medicinal agents.

Park et al. reported that ethyl acetate extract of ginseng berry decreased the fasting blood glucose levels of high-fat diet-induced diabetes mellitus mice, improved hyperglycemia [20], and effectively inhibited the acetylcholinesterase activity and malondialdehyde levels of diabetes mellitus mice brain tissues. Ginsenoside Re was reported as a major compound of ginseng berry ethyl acetate extract, but they did not show the content of ginsenoside Re in ethyl acetate extract. Furthermore, Kim et al. found that ginsenoside Re improved high-fat diet-induced insulin resistance by ameliorating hyperglycemia [21]. The anti-hyperglycemic effect was demonstrated by intragastric administration to mice at a dose of 5 to 20 mg/kg, which is equivalent to 24.33 to 97.3 mg/60 kg administered to humans. Therefore, assuming that the optimal dose of ethyl acetate extract by Park et al. is 20 mg/kg to 50 mg/kg, ethyl acetate extract should contain at least 10 to 40% (*w*/*w*) ginsenoside Re.

Phenolics are everywhere in plant-based foods. Intake of vegetables, fruits, and crops is related to curative benefits against chronic diseases [22]. Phenolics are thought to be responsible for those health effects; it has been suggested that phenolics play an important role in the prevention of many chronic diseases due to their antioxidant, anti-inflammatory, and anti-cancer activities [23]. Oils containing polyphenols, such as olive oils, also have antioxidant, anti-inflammatory, anti-proliferative, pro-apoptotic, neuroprotective, and bone regeneration activities against heart disease, neurodegenerative disease, and cancer [24]. However, in a tour study, ginsenosides rather than phenolic compounds were the main anti-inflammatory biomarkers of GBJ.

Ginsenosides are special compounds, with a steroid glycoside structure, among the phenolic compounds. This is mainly found in the genus Panax. Ginsenosides have been used in traditional medicine due to their various biological effects. Over 30 ginsenosides have been classified into two categories: (1) the 20(S)-protopanaxadiol (PPD) (Rb1, Rb2, Rb3, Rc, Rd, Rg3, Rh2, Rs1) and (2) the 20(S)-protopanaxatriol (PPT) (Re, Rf, Rg1, Rg2, Rh1) [25].

Ginsenoside is absorbed via sodium-dependent glucose co-transporter 1 in the intestines [26]. The bioavailability of ginsenosides has been reported to be extremely low [27,28]. For example, only 3.29% Rg1 and 0.64% Rb1 are absorbed in rat serum after oral administration of ginsenosides [29,30]. Therefore, to maximize the bioavailability and efficacy of ginsenosides, it is necessary to develop ginsenoside-rich sources or to maximize absorption through the improvement of pharmaceutical preparation processes.

The analyzed ginsenosides were reported to have anti-ulcerative and anti-inflammatory effects. Chlorogenic acid, caffeic acid, and coumaric acid were also reported to have anti-ulcerative effects [31,32], but they were present in trace amounts in GBJ and therefore could not be considered as main biomarkers.

In the present study, ginsenoside Rb3 (2.55%), Rc (2.12%), Rd (2.73%), and Re (5.21%) were quantified as the main biomarkers of GB juice, and chlorogenic acid, caffeic acid, and coumaric acid were identified as minor biomarkers (Figure 5). The ginsenosides Rb3, Rc, Rd, and Re in GB juice are thought to be the main factors for relieving acute ulcers *in vivo*, in a mouse model. Ginsenoside Re is thought to be the main component of GB juice, and Re showed the ability to inhibit NF-κB activation when administered orally at 20 mg/kg in a TNBS-induced mouse colitis model [33].

Ginsenoside Rc and Rd have also been reported as anti-inflammatory compounds. Yu et al. reported that oral administration of 5 and 20 mg/kg of Ginsenoside Rc on a HCl/EtOH-induced gastritis mouse model resulted in significant reduction of gastritis symptoms [34]. Yang et al. reported that ginsenoside Rd (10–40 mg/kg) showed anti-inflammatory effects by lowering MPO and proinflammatory cytokine levels and inhibiting phosphorylation of p38 and JNK in a TNBS-induced rat colitis model [35].

Ginsenoside Rb3 has been reported to have an anti-inflammatory effect through TLR4 pathway regulation in LPS-induced macrophage [4]. However, the anti-inflammatory activity of ginsenoside Rb3 in animal models is unknown. Thus, we could not calculate the optimal dose of ginsenoside Rb3 for inflammation control [4].

GB juice was confirmed to have excellent anti-inflammatory activity when administered orally from 250 mg/kg in an acute ulcerative mouse model. Based on the previous reports, it was determined that 250 mg/kg dose of GB juice was the same as 13 mg/kg of Re, 5.3 mg/kg of Rc, and 6.8 mg/kg of Rd orally, and it was thought that the anti-inflammatory activity resulted from the mixed effect of the three ginsenosides. Although it is not possible to calculate the dose of ginsenoside Rb3 *in vivo*, it is thought that the anti-inflammatory effect of Rb3 in vitro can confirm consistent results even *in vivo*. Future studies on the anti-inflammatory activity of Rb3 in in-vivo models are required.

In the present study, GB was obtained when the ginsenoside content was optimal, and the acute inflammation inhibitory effect of GBJ was evaluated without an extraction process using hot water and an organic solvent.

Extraction and concentration are the most important steps in the production of functional materials, and in general, hot water and organic solvent extraction are representatives. Hot water extraction is usually performed using water for four hours or more, followed by hot air, spray drying, and freeze-drying processes. Organic solvent extraction uses ethanol, butanol, ethyl acetate, etc.; however, the safety of the facility must be secured in the extraction and concentration process, and there is a problem with residual solvent after concentration.

GBJ is a functional food material, and Human DAI was calculated as 1.216 g/human/day. Since the daily dose of the GBJ dried product exceeds 1 g, it was thought that the product would be beneficial when manufactured as a semi-solid or liquid formulation. We selected cabbage (CB) as a control and compared it with GBJ. Cabbage is well known for its excellent gastric protective effects [36], and is usually produced as a liquid beverage or tablet using highly concentrated ingredients. CB used in this study was used as a control by freeze-drying a hot water extract product. When administered with CB (500 mg/kg), the rate of gastric damage was about 70%, and when administered with GBJ (250 and 500 mg/kg), inhibition rates of 83% and 92%, respectively, were observed. Therefore, the optimal dose of GBJ could be predicted to be approximately half that of CB (500 mg/kg). The yield of GBJ was 59% and the yield of CB was 12%. GBJ extraction involves a simple juice extraction method, and CB is extracted using a hot water extraction method. If the production method is simply considered, the productivity of GBJ during industrial application is thought to be about 10 times higher than that of CB. In addition, in this study, 4-year-old GB was used to analyze the GB components, and based on the pharmacological efficacy and optimal dosage of GB analysis, it is thought that a good anti-ulcer effect can be obtained when GB is consumed as raw material rather than after complicated extract processing.

## 5. Conclusions

In conclusion, we found that ginseng berry is effective for treating acute gastritis. GBJ inhibited inflammation in a dose-dependent manner in an acute ulcerative mouse model. In a mouse model of acute gastritis induced with HCl/ethanol, ginseng berry juice (GBJ, 250 mg/kg) showed similar gastric injury inhibitory effects as cabbage water extract (CB, 500 mg/kg, P.O). GBJ dose-dependently modulated the pro-inflammatory cytokines such as Tumor Necrosis Factor-α (TNF-α), Interleukin-6 (IL-6), and Interleukin-13 (IL-13). GBJ inhibited the activation of Nuclear Factor kappa bB (NF-κB) and suppressed the expressions of cyclooxigenase-2 (COX-2) and prostaglandin 2 (PGE_2_). In the present study, it was suggested that GBJ is superior to cabbage extract in terms of anti-inflammatory efficacy and productivity.

## Figures and Tables

**Table 1:** The gradient program for the chromatographic method.

	Time	% A	% B
Conditions	0	10	90
	3	12	88
	6	14	86
	9	16	84
	12	18	82
	32	26	74
	40	36	64
	50	50	50
	55	100	0
	57	10	90
	60	10	90

**Table 2:** Biomarker Contents in GBJ.

Biomarker	Content (%)
Ginsenoside rb3	2.55 ± 0.04
Ginsenoside re	5.21 ± 0.21
Ginsenoside rc	2.12 ± 0.16
Ginsenoside rd	2.73 ± 0.02
Chlorogenic acid	0.16 ± 0.03
Caffeic acid	0.07 ± 0.001
*p*-Coumaric acid	0.03 ± 0.001

## Data Availability

Should any raw data files be needed, they are available from the corresponding author upon reasonable request. The data are not publicly available due to some of the technologies in the paper may be relevant to the commercialization process.

## References

[1] Dey L., Zhang L., Yuan C.S. (2002). Anti-diabetic and anti-obese effects of ginseng berry extract: Comparison between intraperitoneal and oral administrations. Am. J. Chin. Med..

[2] Attele A.S., Zhou Y.P., Xie J.T., Wu J.A., Zhang L., Dey L., Pugh W., Rue P.A., Polonsky K.S., Yuan C.S. (2002). Antidiabetic effects of *Panax ginseng* berry extract and the identification of an effective component. Diabetes.

[3] Byun J., Kim S.K., Ban J.Y. (2021). Anti-Inflammatory and Anti-Oxidant Effects of Korean Ginseng Berry Extract in LPS-Activated RAW264.7 Macrophages. Am. J. Chin. Med..

[4] Xu H., Liu M., Chen G., Wu Y., Xie L., Han X., Zhang G., Tan Z., Ding W., Fan H. (2022). Anti-Inflammatory Effects of Ginsenoside Rb3 in LPS-Induced Macrophages Through Direct Inhibition of TLR4 Signaling Pathway. Front. Pharmacol..

[5] Lee K.W., Jung S.Y., Choi S.M., Yang E.J. (2012). Effects of ginsenoside Re on LPS-induced inflammatory mediators in BV2 microglial cells. BMC Complement. Altern. Med..

[6] Nam Y., Ko S.K., Sohn U.D. (2019). Hepatoprotective effect of ultrasonicated ginseng berry extract on a rat mild bile duct ligation model. J. Ginseng Res..

[7] Xu X.Y., Yi E.S., Kang C.H., Liu Y., Lee Y.G., Choi H.S., Jang H.B., Huo Y., Baek N.I., Yang D.C. (2021). Whitening and inhibiting NF-κB-mediated inflammation properties of the biotransformed green ginseng berry of new cultivar K1, ginsenoside Rg2 enriched, on B16 and LPS-stimulated RAW 264.7 cells. J. Ginseng Res..

[8] Xie J.T., Wang C.Z., Ni M., Wu J.A., Mehendale S.R., Aung H.H., Foo A., Yuan C.S. (2007). American ginseng berry juice intake reduces blood glucose and body weight in ob/ob mice. J. Food Sci..

[9] Hu J.R., Chun Y.S., Kim J.K., Cho I.J., Ku S.K. (2019). Ginseng berry aqueous extract prevents scopolamine-induced memory impairment in mice. Exp. Ther. Med..

[10] Kim M.H., Lee J., Jung S., Kim J.W., Shin J.H., Lee H.J. (2017). The involvement of ginseng berry extract in blood flow via regulation of blood coagulation in rats fed a high-fat diet. J. Ginseng Res..

[11] Shin J.E., Jeon S.H., Lee S.J., Choung S.Y. (2022). The Administration of *Panax ginseng* Berry Extract Attenuates High-Fat-Diet-Induced Sarcopenic Obesity in C57BL/6 Mice. Nutrients.

[12] Song S.Y., Park D.H., Seo S.W., Park K.M., Bae C.S., Son H.S., Kim H.G., Lee J.H., Yoon G., Shim J.H. (2019). Effects of Harvest Time on Phytochemical Constituents and Biological Activities of *Panax ginseng* Berry Extracts. Molecules.

[13] Lee S.Y., Cho S.S., Li Y., Bae C.S., Park K.M., Park D.H. (2020). Anti-inflammatory Effect of Curcuma longa and Allium hookeri Co-treatment via NF-κB and COX-2 Pathways. Sci. Rep..

[14] Baeg I.H., So S.H. (2013). The world ginseng market and the ginseng (Korea). J. Ginseng Res..

[15] Kim M., Yi Y.S., Kim J., Han S.Y., Kim S.H., Seo D.B., Cho J.Y., Shin S.S. (2018). Effect of polysaccharides from a Korean ginseng berry on the immunosenescence of aged mice. J. Ginseng Res..

[16] Nam Y., Bae J., Jeong J.H., Ko S.K., Sohn U.D. (2018). Protective effect of ultrasonication-processed ginseng berry extract on the D-galactosamine/lipopolysaccharide-induced liver injury model in rats. J. Ginseng Res..

[17] Jung J., Seo Y.W., Choi J.Y., Kim S.H. (2016). Vestibular function is associated with residual low-frequency hearing loss in patients with bi-allelic mutations in the SLC26A4 gene. Hear. Res..

[18] Lee S.-Y., Jeong J.-J., Eun S.-H., Kim D.-H. (2015). Anti-inflammatory effects of ginsenoside Rg1 and its metabolites ginsenoside Rh1 and 20(S)-protopanaxatriol in mice with TNBS-induced colitis. Eur. J. Pharmacol..

[19] Jang H.-J., Han I.-H., Kim Y.-J., Yamabe N., Lee D., Hwang G.S., Oh M., Choi K.-C., Kim S.-N., Ham J. (2014). Anticarcinogenic Effects of Products of Heat-Processed Ginsenoside Re, a Major Constituent of Ginseng Berry, on Human Gastric Cancer Cells. J. Agric. Food Chem..

[20] Park C.H., Park S.K., Seung T.W., Jin D.E., Guo T., Heo H.J. (2015). Effect of Ginseng (*Panax ginseng*) Berry EtOAc Fraction on Cognitive Impairment in C57BL/6 Mice under High-Fat Diet Inducement. Evid.-Based Complement. Altern. Med. eCAM.

[21] Kim J.M., Park C.H., Park S.K., Seung T.W., Kang J.Y., Ha J.S., Lee D.S., Lee U., Kim D.O., Heo H.J. (2017). Ginsenoside Re Ameliorates Brain Insulin Resistance and Cognitive Dysfunction in High Fat Diet-Induced C57BL/6 Mice. J. Agric. Food Chem..

[22] GutiErrez-Grijalva E.P., Ambriz-Pere D.L., Leyva-Lopez N., Castillo-Lopez R.I., Heiedia J.B. (2016). Review: Dietary phenolic compounds, health benefits and bioaccessibility. Arch. Latinoam. Nutr..

[23] Bondia-Pons I., Aura A.-M., Vuorela S., Kolehmainen M., Mykkänen H., Poutanen K. (2009). Rye phenolics in nutrition and health. J. Cereal Sci..

[24] Garcia-Martinez O., Ruiz C., Gutierrez-Ibanez A., Illescas-Montes R., Melguizo-Rodriguez L. (2018). Benefits of Olive Oil Phenolic Compounds in Disease Prevention. Endocr. Metab. Immune Disord. Drug Targets.

[25] Leung K.W., Wong A.S. (2010). Pharmacology of ginsenosides: A literature review. Chin. Med..

[26] Xie H.T., Wang G.J., Chen M., Jiang X.L., Li H., Lv H., Huang C.R., Wang R., Roberts M. (2005). Uptake and metabolism of ginsenoside Rh2 and its aglycon protopanaxadiol by Caco-2 cells. Biol. Pharm. Bull..

[27] Karikura M., Tanizawa H., Hirata T., Miyase T., Takino Y. (1992). Studies on absorption, distribution, excretion and metabolism of ginseng saponins. VIII. Isotope labeling of ginsenoside Rb2. Chem. Pharm. Bull. (Tokyo).

[28] Xu Q.F., Fang X.L., Chen D.F. (2003). Pharmacokinetics and bioavailability of ginsenoside Rb1 and Rg1 from Panax notoginseng in rats. J. Ethnopharmacol..

[29] Han M., Fang X.L. (2006). Difference in oral absorption of ginsenoside Rg1 between in vitro and *in vivo* models. Acta Pharmacol. Sin..

[30] Han M., Sha X., Wu Y., Fang X. (2006). Oral absorption of ginsenoside Rb1 using in vitro and *in vivo* models. Planta Med..

[31] Shimoyama A.T., Santin J.R., Machado I.D., De Oliveira e Silva A.M., De Melo I.L., Mancini-Filho J., Farsky S.H. (2013). Antiulcerogenic activity of chlorogenic acid in different models of gastric ulcer. Naunyn-Schmiedebergs Arch. Pharmacol..

[32] Boeing T., Costa P., Venzon L., Meurer M., Mariano L.N.B., França T.C.S., Gouveia L., De Bassi A.C., Steimbach V., De Souza P. (2021). Gastric healing effect of p-coumaric acid isolated from Baccharis dracunculifolia DC on animal model. Naunyn-Schmiedebergs Arch. Pharmacol..

[33] Lee I.A., Hyam S.R., Jang S.E., Han M.J., Kim D.H. (2012). Ginsenoside Re ameliorates inflammation by inhibiting the binding of lipopolysaccharide to TLR4 on macrophages. J. Agric. Food Chem..

[34] Yu T., Rhee M.H., Lee J., Kim S.H., Yang Y., Kim H.G., Kim Y., Kim C., Kwak Y.S., Kim J.H. (2016). Ginsenoside Rc from Korean Red Ginseng (*Panax ginseng* C.A. Meyer) Attenuates Inflammatory Symptoms of Gastritis, Hepatitis and Arthritis. Am. J. Chin. Med..

[35] Yang X.L., Guo T.K., Wang Y.H., Huang Y.H., Liu X., Wang X.X., Li W., Zhao X., Wang L.P., Yan S. (2012). Ginsenoside Rd attenuates the inflammatory response via modulating p38 and JNK signaling pathways in rats with TNBS-induced relapsing colitis. Int. Immunopharmacol..

[36] Carvalho C.A., Fernandes K.M., Matta S.L., Silva M.B., Oliveira L.L., Fonseca C.C. (2011). Evaluation of antiulcerogenic activity of aqueous extract of Brassica oleracea var. capitata (cabbage) on Wistar rat gastric ulceration. Arq. Gastroenterol..

